# Malignant pleural effusion diagnosis and therapy

**DOI:** 10.1515/biol-2022-0575

**Published:** 2023-02-28

**Authors:** Liangliang Yang, Yue Wang

**Affiliations:** Department of Thoracic Surgery, China-Japan Union Hospital of Jilin University, No. 126 Xiantai Street, Erdao District, Changchun 130033, China

**Keywords:** malignant pleural effusion, mechanism, diagnosis, therapy, treatment

## Abstract

Malignant pleural effusion (MPE) is a serious complication of advanced tumor, with relatively high morbidity and mortality rates, and can severely affect the quality of life and survival of patients. The mechanisms of MPE development are not well defined, but much research has been conducted to gain a deeper understanding of this process. In recent decades, although great progress has been made in the management of MPE, the diagnosis and treatment of MPE are still major challenges for clinicians. In this article, we provide a review of the research advances in the mechanisms of MPE development, diagnosis and treatment approaches. We aim to offer clinicians an overview of the latest evidence on the management of MPE, which should be individualized to provide comprehensive interventions for patients in accordance with their wishes, health status, prognosis and other factors.

## 1 Introduction

Malignant pleural effusion (MPE) is a pleural effusion (PE) that is caused by a malignant tumor originating in the pleura or by a metastatic malignant tumor from another site that has invaded the pleura [1]. Almost all advanced malignant tumors can invade the pleura and cause MPE. Among the tumors that cause MPE, adenocarcinoma is the most common pathological type, and lung cancer is the most common cause, leading to approximately 1/3 of all MPEs, followed by breast cancer, lymphoma and malignant mesothelioma [2]. Lung cancer is currently the most prevalent malignancy with the highest morbidity and mortality rates globally, and 50–60% of lung cancer patients already have advanced disease at the time of diagnosis. Approximately 10–15% of patients with advanced lung cancer at the initial diagnosis are affected by MPE [3], and the incidence of MPE among re-diagnosed patients is even higher, at more than 50% [4]. Each year, there are one hundred and fifty thousand new cases of MPE in the USA and one hundred thousand in Europe [2,5]. The development of MPE indicates that the tumor has spread or progressed to an advanced stage, and significantly shortening the patients’ survival. The median survival duration of patients at the first diagnosis of MPE is only 3–12 months [6].

In recent decades, research on the mechanisms of MPE has progressed, but there is a lack of effective diagnostic methods and treatments. The management of MPE is currently focused on relieving the symptoms of dyspnea, but the efficacy of such approaches is poor, and patients experience a high recurrence rate and many adverse effects. As molecular targeted therapies have been developed, new diagnostic procedures and treatments are available for patients with advanced tumors. Therefore, it is meaningful to explore new diagnostic and therapeutic treatments for MPE to enhance the quality of life and prolong the survival of patients with MPE. In this article, we review the mechanisms of MPE development, and progress made in diagnosis and treatment techniques.

## 2 Mechanism and composition of MPE

**Figure 1:**
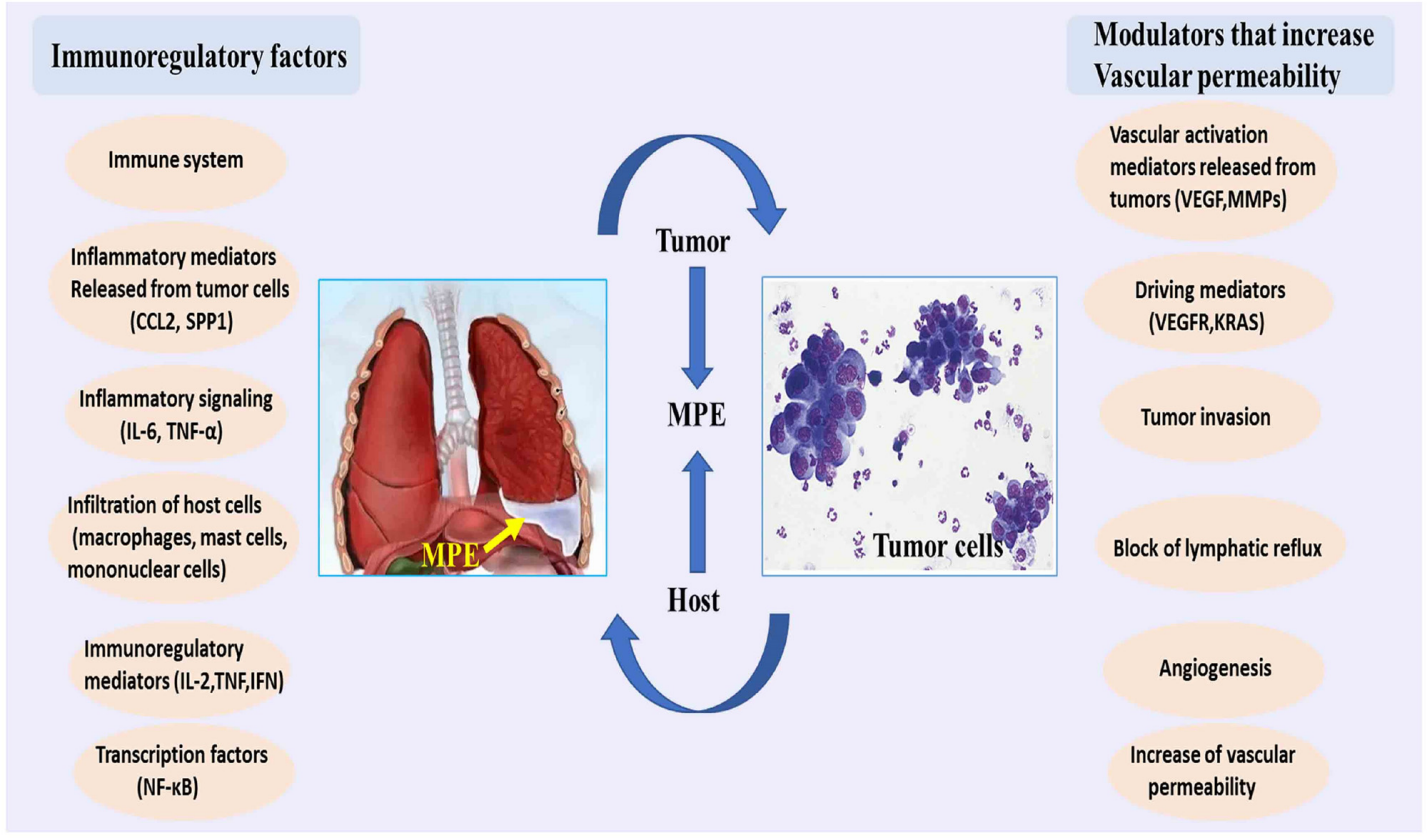
Pathogenesis of MPE. Many effector molecules from host cells or tumor cells are involved in the pathogenesis of MPE. These effectors can generally be divided into two categories. The first kind of effector molecules are important immune regulatory factors, including IL-2, TNF and interferons (IFNs). The second group of effector molecules is an effective regulator to increase vascular permeability, including VEGF, MMPs and many other molecules. CCL, IL, KRAS, MMPs, TNF; VEGF and VEGFR.

In the past few years, the mechanisms of MPE development have been increasingly studied and have shifted from a single factor, initially considered to be a blockage of lymphatic return, in favor of a combination of multiple factors, even down to the level of molecular mechanisms. A variety of cells are present in the pleural cavity microenvironment, including host cells (e.g., pleural mesothelial cells and endothelial cells) as well as cells of myeloid origin and the lymphatic system, which interact with tumor cells to accelerate angiogenesis and increase vascular permeability and inflammatory responses in tumor tissues, ultimately leading to the development of MPE [7,8]. MPE is the result of integrated interactions between host and tumor cells, as summarized by Stathopoulos and Kalomenidis [7]. Many effector molecules, from either the host or tumor cells, are involved in its pathogenesis. These effectors can generally be classified into two categories, including immunoregulatory effectors and modulators, which increase vascular permeability (Figure 1). The immunoregulatory factors include interleukin (IL)-2, tumor necrosis factor (TNF) and interferons. Important regulators of the induction of vascular permeability are vascular endothelial growth factor (VEGF), matrix metalloproteinases (MMPs) [9]. Among these effector regulators, VEGF plays a central role in PEs [10].

MPE is mostly hematogenous, massive, rapidly, growing composed of lymphocytes with protein content more than 30 g/L and LDH more than 500 U/L [11,12]. Approximately 40–90% of MPEs have been found to contain malignant tumor cells. Malignant tumor cells in MPE often have enlarged nuclei of different sizes, aberrant nuclei, and hyperchromatic nuclei. If the range of pleural lesions is widespread, it is difficult for glucose and acidic metabolites to penetrate the pleura. Both glucose and pH are low, indicating that the tumor is widely infiltrated and the patient’s survival time is short [13,14].

### 2.1 Tumor angiogenesis and increased vascular permeability

Tumor angiogenesis and increased vascular permeability are vital factors in the formation of MPE. VEGF has been found to maintain endothelial cell survival through tyrosine phosphorylation of focal adhesion kinase, which plays a key role in the chemotactic response of VEGF and can also influence angiogenic signaling pathways, unlike in benign PE [10,15]. VEGF is mainly mediated by vascular endothelial growth factor receptor 2 (VEGFR-2), which promotes increased vascular permeability by disrupting endothelial cell integrity, disrupting junctions and increasing cellular gaps [16]. Angiopoietin 1/2, a factor secreted by tumor cells, also has a role in promoting increased vascular permeability [17]. In addition, it has been shown that mast cells have a significant effect on the formation of MPE and that the release of trypsin-α/β1 and interleukin-1β increases pulmonary vascular permeability and induces the activation of inhibiting nuclear transcription factor (NF-κB) in the pulmonary vasculature, promoting fluid accumulation and tumor growth [18]. The above findings provide new directions for the treatment of MPE, and therapeutic approaches targeting increased tumor angiogenesis and permeability, such as a recombinant human vascular endothelial inhibitors and VEGF monoclonal antibodies, have improved efficacy in the treatment of MPE.

### 2.2 Immune microenvironment

Due to the increasing interest in the cellular microenvironment and immune responses, there has been increasing attention on the immune microenvironment of the MPE locus, including the interaction of immune cells, cytokines and tumor cells, which together form a microenvironment that promotes MPE development. Mononuclear macrophages are the main immunoinflammatory cells involved in MPE formation, and CD14^+^ macrophages are specifically highly expressed in MPE and therefore can serve as an immunodiagnostic marker for MPE [19]. Principe et al. showed that autocrine Interleukin-6 (IL-6) from tumor cells promoted MPE development by inducing activation of the Stat3 pathway and upregulating VEGF expression [20]. In addition, the number of Th1, Th17, Th9 and Th22 cells, which also play a role in the PE microenvironment by secreting cytokines, was increased in MPE compared to in peripheral blood [21]. In addition, chemokine (CC motif) ligand 20 (CCL20) and CCL22 can recruit peripheral blood Th17 cells to infiltrate the pleural space [22]. Thus, an imbalance of immune cells can also induce angiogenesis, increase vascular permeability, and promote the development of tumor metastasis and inflammatory responses, ultimately fostering the formation of MPE.

## 3 MPE diagnosis

### 3.1 Chest ultrasonography

Ultrasound of the chest is recommended to examine PE, and is an accurate indicator of the volume and depth of PE, the presence of contralateral PE, echogenicity, compartmentalization, intracavitary effusion, presence of pleural thickening and nodules, and diaphragm position and motion [23]. Metastatic pleural tumors show the typical ultrasound findings of relatively small hypoechoic crystalloid-like masses, blunt chest wall margins, or masses or nodules with complex echogenic intensity [24]. Only 9% of patients with nonmalignant PE present with this phenomenon. Another retrospective study analyzing the ultrasound characteristics of 104 patients with MPE found that 95 (91.35%) had nonmalignant PE, and nine (8.65%) had MPE, additionally the pleural nodules with intercostal wall thickening >10 mm and diaphragmatic thickening >7 mm were significant predictors of MPE on ultrasound (sensitivity 73% and specificity 100%) [25]. Both studies indicated that on ultrasound, pleural thickening, nodules and non-encapsulated effusions are reliable indicators for diagnosing MPE. The use of ultrasound guidance during pleural surgery can result in greater success rates and a reduced risk of complications, including parenchymatous organ injury. Ultrasound guidance also provides valuable information about the accessibility of the PE sac, and Doppler imaging helps to localize and avoid intercostal vessel injury prior to pleural intervention, contributing to a reduction in complications [23].

### 3.2 Computed tomography (CT)

Chest CT can be useful in identifying benign and malignant pleural diseases, including through signs such as thickening of the wall pleura or mediastinal pleura greater than 1 cm. Irregular pleural cavities and pleural nodules suggest a high probability of MPE with a high specificity (88–94%) but low sensitivity (36–51%), and approximately 50% of patients with MPE show no pleural abnormalities on CT [26,27]. Chest CT should be performed before the fluid is completely drained so that the pleural phase contrast can be used to obtain better clarity to better visualize pleural abnormalities [28]. Mediastinal and pleural thickening and pleural nodules suggest a possible MPE [27]. Although CT scans may not necessarily reveal abnormalities other than PE, CT scans can also provide evidence of underlying primary tumor and metastases.

### 3.3 Positron emission tomography-computed tomography (PET-CT)

The fluorodeoxyglucose PET imaging is based on the different metabolism between normal and abnormal tissues, in which the metabolism of fluorodeoxyglucose is accelerated and reflected by higher uptake in tumor cells; however, this method has a false-positive rate in the presence of metabolically active inflammation and infection [29]. Although its popularity influenced by false-positive results for inflammation and infection, PET-CT can be applied to stage pleural disease [30]. Nonetheless, one study showed that PET-CT can identify benign and MPE with a sensitivity of 83.3% and specificity of 92.2% and developed and validated a PET-CT parameter score, which can help physicians distinguish PE [31].

### 3.4 Cytology

The criteria for the diagnosis of MPE are the detection of cancer cells by pleural fluid cytology or the detection of cancer cells by pleural biopsy. Cytological diagnosis is used as a traditional diagnostic tool, but its sensitivity is not high if there are few tumor cells [32]. Several studies have shown that the sensitivity of pleural fluid cytology is approximately 40–87%, with low positive rates. The volume of cytological fluid extracted by diagnostic thoracentesis is currently controversial, and some studies have shown that at least 75 mL of pleural fluid is required for cytopathological diagnosis [15]. The sensitivity of a pleural fluid cytology sample taken in the first thoracentesis for the diagnosis of malignancy is 60%, with an increased sensitivity of 27% in the second procedure, but after the third, the diagnostic rate no longer increases, and the additional procedure instead delays diagnosis and treatment [33]. The use of cytology in combination with pleural biopsy increases the sensitivity of the diagnosis [34]. Although pleural fluid cytology is the least invasive, slowest and least effective method for diagnosing malignancy, the cytological diagnosis is also dependent on the underlying primary tumor, sample preparation and the experience of the cytologist [1].

### 3.5 Pleural biopsy

Pleural biopsy is the gold standard for the diagnosis of MPE [6]. In CT-guided or ultrasound-guided biopsies can be performed to collect pleural tissue for diagnosis, and this method has a sensitivity of 76–88% and specificity of up to 100% for the diagnosis of MPE [6]. When pleural thickening >1 cm is found in MPE, the diagnostic sensitivity of CT-guided pleural biopsy is comparable to that of thoracoscopy (96 versus 95%) [35]. Pleural biopsy in addition to of pleural fluid cytology can increase the diagnostic sensitivity for MPE by 7–27% [27]. When pleural thickening is obvious, pleural biopsy via thoracoscopic surgery can help simultaneously visualize the pleura, biopsy the involved area and drain the effusion. In addition, if the pleura is infiltrated and there is no trapped lung, pleural fixation can be performed [23].

### 3.6 Medical thoracoscopy (MT)

MT is both a diagnostic and a therapeutic method, and this method has a sensitivity of 92–97% and a specificity of 99–100% for the diagnosis of MPE [36,37]. The indications for MT, from a diagnostic point of view, include: cytologically negative and predominantly lymphocytic exudate, and the need for tumor staging, for additional tissue for molecular analysis, and to identify lung cancer, breast cancer and mesothelioma; from a therapeutic point of view, the goals of MT include pleural fixation by spraying in talcum powder, and drainage of MPE, especially complex pneumonia-like MPE, and the absolute contraindications, include extensive adhesions of the lung to the mural pleura, hypercapnia, severe dyspnea, uncontrollable cough, and myocardial infarction within the last 4 weeks [38]. Endoscopic thoracoscopy is a safe procedure with low complications and mortality rates and high diagnostic performance. Procedures requiring general anesthesia are very useful if more invasive procedures, such as video-assisted thoracic surgery (VATS), are performed for patients considered unsuitable for surgery or who are at increased risk for complications [39,40]. These procedures (medical or surgical thoracoscopy) have similar performances, with low mortality and major complication rates (between 0–0.34% and 1.2–1.8%, respectively [41,42].

### 3.7 Liquid biopsy

Gene expression of different tumors has become a pillar of diagnosis, therapeutic monitoring and precise medical guidelines. Numerous studies on liquid biopsies have demonstrated this diagnostic utility of this method in diagnosing disease, predicting prognosis and detecting cell-free deoxyribonucleic acid (DNA) in plasma [43]. MPE is often enriched with tumor cells, and extracellular vesicles and cell-free DNA are the two main targets currently explored in MPE. Therefore, MPE can be used as a source of biomarkers for the study of tumor mutations in liquid biopsies. Compared to tissue biopsy, the liquid biopsy method is less invasive and can show dynamic changes in the tumor in real time by using circulating biomarkers, including cell-free DNA, circulating tumor DNA, extracellular vesicles, messenger ribonucleic acid (mRNA), micro-ribonucleic acid (miRNA), circulating tumor cells and exosomes [44–46]. With the continuous development of detection technologies, multigene sequencing and second-generation sequencing (NGS) methods to detect the differences between mutations in pleural fluid and tissue specimens are also a hot topic of research [47,48]. Lin et al. simultaneously collected pleural fluid and plasma specimens from 63 patients and extracted free DNA and precipitated cell-free DNA from the pleural fluid supernatant and cell precipitate, respectively. All samples were analyzed through received NGS for 416 cancer-associated genes and showed that in cohort 1 with matched tumor tissues, 93.1% of tissue-assayed driver mutations were detected in MPE cell-free DNA, including activin-like kinase (ALK), v-raf murine sarcoma viral oncogene homolog B1, EGFR, kirsten rats arcomaviral oncogene homolog (KRAS), neuro kinin, neurofibromin 1, phosphatidylinositol-4,5-bisphosphate 3-kinase catalytic subunit alpha and proto-oncogene tyrosine-protein kinase receptor ret, whereas only 62.1% were detected in plasma cell-free DNA [49]. PE cell-free DNA had the highest detection rate of EGFR mutations in the entire cohort (71% overall, 68 and 59% for PE cell-free DNA and plasma cell-free DNA, respectively) [50]. In bleeding or cytology-negative PE samples, PE cell-free DNA had a higher sensitivity for mutation detection than plasma cell-free DNA. This suggests that MPE sampling could be a potential alternative to liquid biopsies and that genetic analysis of disease by detecting circulating tumor cells and exosomes in PEs could be explored in the future [51,52]. In addition, the different sensitivities and specificities of different sequencing platforms still require further study in clinical studies and standardization and are worthy of further investigation in the future.

### 3.8 MPE diagnostic summary

The sensitivity of the first PE cytology sample to diagnose malignant tumors was 60%, and the sensitivity of the second PE cytology sample increased by 27% [53]. However, after the third pleural puncture, the diagnostic rate would not increase, and the diagnosis and treatment would be delayed. Therefore, when the second cytological examination is negative yet MPE is highly suspected, the next step should be imaging guided pleural biopsy or thoracoscopic pleural biopsy. It has been reported that pleural biopsy based on pleural fluid cytology can increase the diagnostic rate of MPE by 7–27% [54]. The sensitivity of ultrasound or CT guided pleural biopsy was higher than that of blind pleural biopsy. The sensitivity of ultrasound guided pleural biopsy in the diagnosis of MPE was 70–94% [54]. Some studies believed that five-eight needles of image guided pleural biopsy have high diagnostic sensitivity, and six needles are recommended at least [53]. The sensitivity and specificity of MT in the diagnosis of MPE were 92–97.0 and 99–100%, respectively, which were similar to those of VATS [36]. The methods for diagnosing MPE and its causes are increasingly updated, which has improved the diagnostic accuracy of MPE to a certain extent, but they still have certain limitations. It is still to be confirmed by prospective studies with large samples to determine whether any better or integrated approach, which can make the diagnosis more accurate in the future.

## 4 Management of MPE

The aim of MPE treatment is to relieve symptoms such as dyspnea, improve quality of life and prolong survival. The British Thoracic Society guidelines for the management of MPE and the American Thoracic Society (ATS) guidelines recommend that patients with MPE should be observed for only if the MPE is asymptomatic [55], and that for symptomatic patients, it should first be determined if their symptoms are related to MPE, and these patients should be assessed for lung distension [4]. Treatments for MPE currently include therapeutic pleurodesis, chemical pleural fixation, thoracoscopic pleural fixation, long-term tube drainage, pleurodesis and thoraco-abdominal shunts. Pleural fixation and thoracic drainage are the two most commonly used methods in clinical practice. It is also worth exploring whether a difference exists in the efficacy of tyrosine kinase inhibit (TKI) therapy for patients with EGFR mutations and MPE and intrathecal therapy in combination with TKI, as the standard first-line treatment option for patients with EGFR mutations [56].

### 4.1 Thoracentesis

Thoracentesis is the most commonly used clinical modality to treat MPE and provides rapid relief of dyspnea symptoms; furthermore, confirmatory evidence of diagnosis can be obtained by thoracentesis in more than two-third of patients [57]. Repeated thoracentesis may be considered for patients with short expected survival duration and those who are unable to tolerate more invasive procedures and treatments, but clinicians need to adequately prevent the various complications that may arise from repeated thoracentesis, such as infection, bleeding and pneumothorax. The timing and scope of thoracentesis depends on the type and location of the primary tumor and the general condition of the patient. In the early stages of MPE, the effusion can be left untreated, but a clear diagnosis is needed as soon as possible, while progressive MPE needs to be treated promptly; otherwise, the patients are prone to developing irreversible pulmonary atelectasis and lung atrophy. Repeat chest CT or X-ray is required after thoracentesis to avoid medically induced pneumothorax or hemothorax [4]. Care should also be taken to avoid the rapid extraction of fluid by thoracentesis. Rapidly extracting more than 1.5 L of fluid from the pleural cavity in a 24 h period may result in redundant pulmonary edema in the affected lung, a complication that has a mortality rate of up to 20%, so it is recommended that each thoracentesis aspiration be limited to 1.0–1.5 L [4]. Successful thoracentesis can provide the patient with a clear diagnosis of MPE, as well as rapid recovery from the compressed lung tissue, which is relevant to the success of subsequent chemical pleural fixation [58].

### 4.2 Pleurodesis

The purpose of pleural fixation is to induce an inflammatory response in the pleura, forcing the two layers of the pleura to adhere in order to prevent fluid accumulation. The main focus of pleural fixation is the choice of sclerosing agents, including chemotherapeutic agents, bio-immunotherapy agents, talc, iodophor, silver nitrate, herbal preparations and autologous blood. Of these, talc is the most widely used and effective pleural fixation agent [23]. A thin flexible catheter is placed under ultrasound or CT guidance and secured to the chest wall, and a drainage bag or water seal bottle is attached to drain the pleural fluid; these devices have the advantage of being easy to operate, safe and not especially painful for the patient. Adequate chest drainage can be followed by the intrapleural application of antitumor drugs (e.g., bleomycin) or nontumor drugs (e.g., talcum powder) as sclerosing agents and adhesives to achieve pleural fixation. As the most commonly used clinical pleural adhesion agent, medical talc has the advantages of a high success rate for pleural fixation (approximately 93%) and low cost [59]. Talc can be injected into the chest cavity not only through a drainage tube, but also as a dry powder that can be sprayed uniformly throughout the chest cavity during surgical thoracoscopy [60]. The most significant side effect of pleural fixation is a local inflammatory response in the pleura leading to fever and chest pain, and pleural inflammation is thought to be directly related to successful pleural fixation, although a recent analysis of the TIME 1 trial dataset showed no difference in pain scores between patients with successful and unsuccessful pleural fixation, and elevated C-reactive protein levels were associated with successful pleural fixation [61]. Other rare complications include local infection, chest sepsis, arrhythmias, cardiac arrest, myocardial infarction and hypotension. Complications such as acute respiratory distress syndrome, acute pneumonia, and respiratory failure have also been occasionally reported [60]. In a multicenter retrospective study, none of the 558 patients who underwent pleural fixation with talc developed acute respiratory distress syndrome [62], and another study showed that pleural fixation with talc resulted in a lower recurrence rate for pleural fluid than pleural fixation with bleomycin (relative risk [RR] 0.64; 95% confidence interval [CI] 0.34–1.20), compared with the use of a talc solution. Spraying dry talc powder resulted in more effective fixation of the pleura and a lower recurrence rate for pleural fluid (RR 0.21; 95% CI 0.05–0.93) than other pleural fixation methods [63]. Nonrandomized studies have shown that patients who underwent successful pleural fixation survived longer than those who did not, which may indicate a biological role for pleural fluid in cancer progression [64]. A recent meta-analysis found no correlation between large and smallbore chest drains for successful pleural fixation between groups of patients [65]. Other factors such as turning the patient after sclerotherapy injection, intermittent open chest tube drainage and the application of multiple chest tube drains did not lead to a difference in fixation outcomes.

### 4.3 Indwelling pleural catheter (IPC)

The IPC is a silicone catheter placed in the pleural cavity and is carried through a subcutaneous tunnel. It has a cuff at the distal end that forms fibrous adhesions in the subcutaneous tissue over time, holding the IPC in place and preventing ascending infection. The IPC is discharged through a vacuum bottle with a one-way valve that uses negative suction to monitor flow and volume. The IPC offers the advantage of outpatient/home management of MPE and has gained great popularity in the last decade due to the reduction in hospital admission time [66–68]. A meta-analysis of 1,348 patients with MPE treated with IPC showed that 95.6% of patients had improved dyspnea symptoms and 45.6% had spontaneous pleural adhesions after a mean of 52 days [69]. Given their ease of insertion and management, IPC has become the management modality of choice in many centers around the world. However, IPC is associated with a higher rate of complications, among which, the complication of greatest concern is pleural infection, particularly in patients receiving chemotherapy [70]. In a large multicenter study of 1,021 patients with IPC, pleural infections occurred in 50 (4.9%) patients, 94% of whom had a mortality rate of less than 0.3%, with the mortalities being associated with the control of pleural infections by antibiotics [71]. In patients with irreversible atelectasis or pulmonary atrophy due to prolonged fluid compression or recurrent massive MPE following chest tube drainage, chest tube drainage may not adequately and effectively drain the pleural fluid; additionally, if the patient requires outpatient treatment or treatment at home with a tube, chest tube drainage may be a suitable alternative to closed chest tube drainage because it requires more medical care (e.g., tube management). However, closed chest drainage can be used as an alternative to chest tube drainage [70,72], allowing patients to be more easily treated in outpatient clinics with easier care [73]. A drain thicker than a subclavian venipuncture tube can be placed subcutaneously for a longer distance, which reduces the chance of infection, and several studies have shown that closed chest drains are safe and effective, with a treatment efficiency of approximately 90% and low rate of long-term complications (including pain, local cellulitis and chest tube dislodgement) [74]. Additionally, approximately 26–58% of patients develop spontaneous pleural fixation and eventually have their chest tubes removed after a period of closed chest drainage [75]. In a single-center retrospective study from the Netherlands, 17 patients with recurrent MPE underwent closed chest drainage, with 70–80% of patients experiencing significant symptomatic relief. The mean indwelling time of the tube was 2.3 months (range 1–6 months), and the mean drainage volume was 360 ml (range 150–1,000 mL) [76]. For patients requiring long-term drainage, the overall results of closed chest drainage are satisfactory and can significantly improve patient quality of life.

### 4.4 Anti-angiogenic agents

In recent years, intracavitary biotherapy has been a hot research topic in MPE treatment. Domestic studies have confirmed that recombinant human vascular endothelial inhibitor combined with the intracavitary administration of chemotherapy can control malignant plasma cavity effusion with a control rate of approximately 60% [77], suggesting the promising application of anti-angiogenic drugs in the treatment of malignant thoracic and abdominal effusion; moreover, combinations with other thoracic drugs (cisplatin, etc.) have improved efficiency compared with single drugs. Bevacizumab is a recombinant humanized monoclonal antibody that binds to VEGF, thereby preventing neovascularization [78]. Du et al. reported that among 72 patients with MPE were randomly assigned to an intracavitary bevacizumab combined with cisplatin group and groups, combination treatment was more effective than treatment with a single agent (83.3 vs 50.0%, *P* < 0.05), leading to prolonged progression-free survival (PFS) 5.3 vs 4.5 months, *P* < 0.05), with a trend toward longer overall survival (OS) [79]. In a study by Tao et al. [80] 21 patients with advanced lung adenocarcinoma with MPE treated with bevacizumab in combination with chemotherapy (paclitaxel in combination with platinum, gemcitabine in combination with platinum and pemetrexed alone) had a median PFS and median OS of 7.8 and 25.8 months, respectively. The MPE control rates at weeks 6, 12, 24, 48 and 96 were 95.2, 90.0, 89.5, 73.7 and 43.8%, respectively. Thus, bevacizumab, whether administered intrathecally or intravenously, may be more effective in controlling MPE than chemotherapy alone.

### 4.5 EGFR-TKI targeted agents

Most non-small cell lung cancer (NSCLC) patients are in advanced stage at the time of diagnosis, and the probability of accepting surgical resection is less than 30% [3]. Among them, patients with EGFR gene mutation and echinoderm microtubule-associated protein-like 4 (EML4-ALK) gene rearrangement can use targeted drug therapy [81,82]. Several studies have now established EGFR-TKI as the treatment of choice in patients with EGFR mutations [83]. It is worth considering whether intrathecal drugs can control MPE patients better with EGFR mutations and MPE, or whether receiving single-agent TKI therapy can control MPE while avoiding the adverse effects of intrathecal drugs such as fever, bone marrow suppression and chest pain. Kashiwabara et al. [56] analyzed the efficacy of EGFR-TKI in combination with talcum powder in patients with MPE and lung adenocarcinoma, 34 of whom received TKI as first-line therapy. It was suggested that TKI-targeted therapy combined with pleural fixation is more effective than pleural fixation alone in controlling MPE in patients with EGFR mutations and MPE. EGFR-TKIs treatment can significantly improve the prognosis of patients with advanced NSCLC, especially those with EGFR gene mutation. However, a retrospective study showed that the response of PE of lung cancer patients with EGFR mutation and MPE to EGFR-TKIs (including gefitinib, alfatinib and oxitinib) was worse than that of solid malignant tumors [84]. In NSCLC patients, anaplastic lymphoma kinase (ALK) gene or ROS proto-oncogene 1, receptor tyrosine kinase (ROS1) rearrangement accounts for 5 and 1%, respectively [85]. They are sensitive to the treatment of coxotinib, seretinib, aletinib and bugatinib. Compared with chemotherapy, all ALK inhibitors can significantly improve the PFS of lung cancer patients in this group. Compared with coxotinib and seretinib, aletinib and bugatinib can better improve the PFS of patients, especially in patients with advanced stage [86,87]. However, there is no large clinical study confirmed that the prognosis of patients with advanced lung cancer with MPE is different from that of patients with advanced lung cancer without MPE.

### 4.6 The thoracoperitoneal shunt

For patients whose lungs cannot be expanded or trapped due to MPE, a thoracoabdominal shunt tube can be placed, which is a subcutaneous tunnel from the chest to the abdomen [88]. The liquid is pumped manually, but due to the limited capacity of the pump chamber, it needs to be pumped frequently [89]. A retrospective study on thoracoperitoneal shunt showed that the remission rate was 95%, but the complication rate was 15%, including technical failure and infection [90]. The presence of ascites is a contraindication of thoracoperitoneal shunt.

### 4.7 Biological agent treatment

The commonly used biological agents in clinic are mainly IL-2 and TNF [91]. IL-2 is a thymus dependent lymphocyte growth factor, which can induce the secretion of interferon and a variety of cytokines, including promoting the long-term survival of T cells, enhancing the killing activity of T cells, promoting the proliferation and activation of natural killer (NK) cells, lymphokine activated killer cells, tumor infiltrating lymphocyte cells, etc. [92]. It is clinically used for tumor adjuvant therapy and the treatment of cancerous PE. Studies have shown that the use of IL-2 alone or in combination with other anti-cancer therapies can bring survival benefits to patients with advanced cancer. TNF-α is a cytokine secreted by monocytes/macrophages [93]. Its anti-tumor mechanisms mainly include directly killing tumor cells to induce tumor cell apoptosis, anti-tumor angiogenesis and enhancing immune function. TNF-α thoracic perfusion therapy for MPE has shown good efficacy whether alone or in combination [94]. The clinical application is limited due to its expensive price, high fever and other side effects.

### 4.8 Immunotherapy

Immunotherapy consists mainly of immune cell therapy and immune checkpoint (programmed death-1/programmed cell death-ligand-1 and cytotoxic T lymphocyte-associated antigen-4) inhibitor therapy [95,96]. Intrathoracic autoimmune cell infusion therapy is an alternative approach to control MPE, and the effectiveness correlates with a higher frequency of peripheral blood effector T cells [97]. Although the efficacy is limited, it is less adverse, well tolerated, and not limited by the patient’s liver or kidney function status or physical status score, and has good promise in the palliative treatment of MPE. For advanced non-squamous NSCLC and squamous lung cancer with negative driver genes, including patients with combined MPE, immune checkpoint inhibitors alone or in combination with chemotherapy and anti-angiogenic drugs can improve patient prognosis [98].

## 5 Future directions

Most of the interest is the early translational work which shows that the proliferation of cancer cell culture is promoted by seeding cells in pleural fluid [99]. This growth-promoting property of pleural fluid opens up the possibility that pleural fluid may not be a bystander to malignant disease, requiring only drainage to relieve symptoms, but may be an active promoter of cancer progression, thus emphasizing the importance of early control of PE. Current treatment strategies are focused on mechanical drainage and pleural cavity sealing [28]; however, significant efforts should be directed toward more complex areas of biomarker analysis and validation, and subsequent treatment with intrapleural immune agents and targeted therapies, for many years to come. If successful, this therapeutic strategy has the potential to bring about real step change in the management of MPE [100]. However, there are significant challenges in this regard, especially the research evidence that the clinical heterogeneity of MPE depends on primary tumors and intrapleural therapy. So far, the results of MPE are mixed.

## 6 Prognosis

Researchers have investigated several predictors of survival in patients with MPE. The most established method for predicting the prognosis of MPE patients is the LENT score [101] which includes the human l-lactate dehydrogenase (L-LDH) level, Eastern Cooperative Oncology Group (E-ECOG), N-neutrophil to lymphocyte ratio, T-tumor type and divides patients into low (score 0–1), moderate (score 2–4) or high (5–7) risk with a median survival of 319, 130 and 44 days. Additionally, as the LENT score (Table 1) has been developed using patients presenting with their first episode of MPE, regardless of previous cancer treatment, it is widely applicable and relevant in the clinical setting. It not only guides the treatment of MPE, but also helps predict the survival of MPE patients. It is the first validated risk stratification system for predicting the survival of MPE patients and is superior to ECOG performance status alone in predicting survival in individual patients.

**Table 1:** The LENT score calculation

		Variable	Score
L -LDH level in pleural fluid (IU/L)		<1,500	0
		>1,500	1
E-ECOG PS		0	0
		1	1
		2	2
		3–4	3
N-NLR		<9	0
		>9	1
T-tumor type	Lowest risk tumor types	Mesothelioma hematological malignancy	0
	Moderate risk tumor types	Breast cancer gynecological cancer	1
	Highest risk tumor types	Renal cell carcinoma	
		Lung cancer other tumors types	2
			Total score
Risk categories		Low risk	0–1
		Moderate risk	2–4
		High risk	5–7

Patients with MPE are a highly diverse group of patients, with great variation in primary tumor involvement (particularly the ability to expand the lungs), major comorbidities, performance status, expected survival and patient wishes. More patient-based studies are likely to be performed in the future. In addition to research studies on pain, dyspnea and quality of life as well as studies on supportive treatments such as exercise training, nutritional interventions and psychological support should be conducted and may play an important role for MPE and may also offer useful adjuncts to interventions for MPE. Adjunctive treatments, such as exercise and dietary interventions, may also be useful adjuncts to pleural interventions [102].

## 7 Conclusions

There have been significant advances in the management of MPE. However, patients with advanced malignancies remain severely ill. Recent discoveries in the pathophysiological mechanisms of MPE have highlighted the role of molecular factors and mutations in the disease. There remains a need for individualized treatment. Predictive scores in clinical practice help to determine prognosis and guide treatment.

The current treatment for MPE is individualized and multidisciplinary. Some patients in poor general condition, especially those with an expected survival of only a few weeks to a few months, typically opt for less invasive methods of pleural fluid control, such as repeated thoracentesis or closed chest drainage, which are ideal for reducing both the length of hospital stay and discomfort of the patient. With the continuous development of treatment techniques and tools, VATS techniques can not only relieve the clinical symptoms associated with MPE but also allow for clear pathological results and direct observation of tumors progression in the thoracic cavity, as well as facilitate various therapeutic operations such as pleurectomy and pleural fixation. Because it is performed under direct vision, surgical thoracoscopic pleural fixation is more uniform and thorough in spraying sclerosing or adhesive agents. This method is therefore more accurate, and a combination of treatments is recommended based on patient’s specific situation. In the near future, improved treatment for oncological disease and a better understanding of the pathophysiological mechanisms of MPE will help to improve the prognosis of patients with MPE. Many unanswered questions remain and ongoing research will help clinicians to enhance the care for this group of patients with MPE.
